# Case Report: Acne related to electric welding, a new type of occupational acne

**DOI:** 10.3389/fmed.2026.1909578

**Published:** 2026-08-18

**Authors:** Renchuan Jia, Sushupti Kayastha, Shixin Han

**Affiliations:** 1 Department of Dermatology, The Second Clinical College of Guangzhou University of Chinese Medicine, Guangzhou, China; 2 Department of Dermatology, First Hospital of Dalian Medical University, Dalian, China

**Keywords:** acne, acneiform lesions, chloracne, electric welding, occupational acne

## Abstract

Occupational dermatoses constitute a significant proportion of occupational diseases, among which occupational acne can cause irreversible scarring and immense psychological distress in affected workers due to its disfiguring nature. Early recognition of suspected ocupational skin diseases is crucial for effective prevention and treatment. Electric welding exposes workers to a complex mixture of metal fumes, ultraviolet radiation, heat and other potential occupational hazards that may contribute to acne development. Here, we report two cases of persistent nodular acne linked to electric welding exposure observed at the First Affiliated Hospital of Dalian Medical University in China. Both patients presented with persistent nodular acne clearly associated with welding environments, with improvement during periods away from occupational exposure. Based on our clinical findings and a review of the potential etiological factors and pathogenic mechanisms, these cases suggest a distinct new subtype of occupational acne related to electric welding hazards. Recognizing these characteristics may help in early detection, prevention and management of occupational dermatoses.

## 1 Introduction

Occupational acne refers to chronic inflammatory lesions of the skin's hair follicle and sebaceous gland system caused by exposure to certain specific occupational hazards such as mineral oils or halogenated aromatic hydrocarbons during production and labor. It is mainly divided into oil acne and chloracne (1). Currently, there are no published literature reports worldwide on acne induced by electric welding. However, in clinical practice, we have frequently encountered acne patients who have a close relationship with electric welding work. We think it is a new subtype of occupational acne called welder's acne. Here, we present two typical cases for a brief report and summarize the characteristics and potential etiological factors of this type of acne, in the hope of drawing sufficient attention to this patient population and enriching the types of occupational skin diseases.

## 2 Case description

### 2.1 Case 1

A 30-year-old male electrical welder presented with acne that appeared on his face shortly after he began working in electrical welding 3 years ago. He reported the eruptions to be particularly severe during the summer months and improved during vacations. He had no history of acne during puberty and neither his siblings nor other family members had a history of acne or other dermatological diseases. He reported no exposure to other industrial or chemical substances in either his occupational or living environment. In addition, he had no history of drug or cosmetic allergies and had not used any special medications or skincare products in recent years.

**Figure 1:**
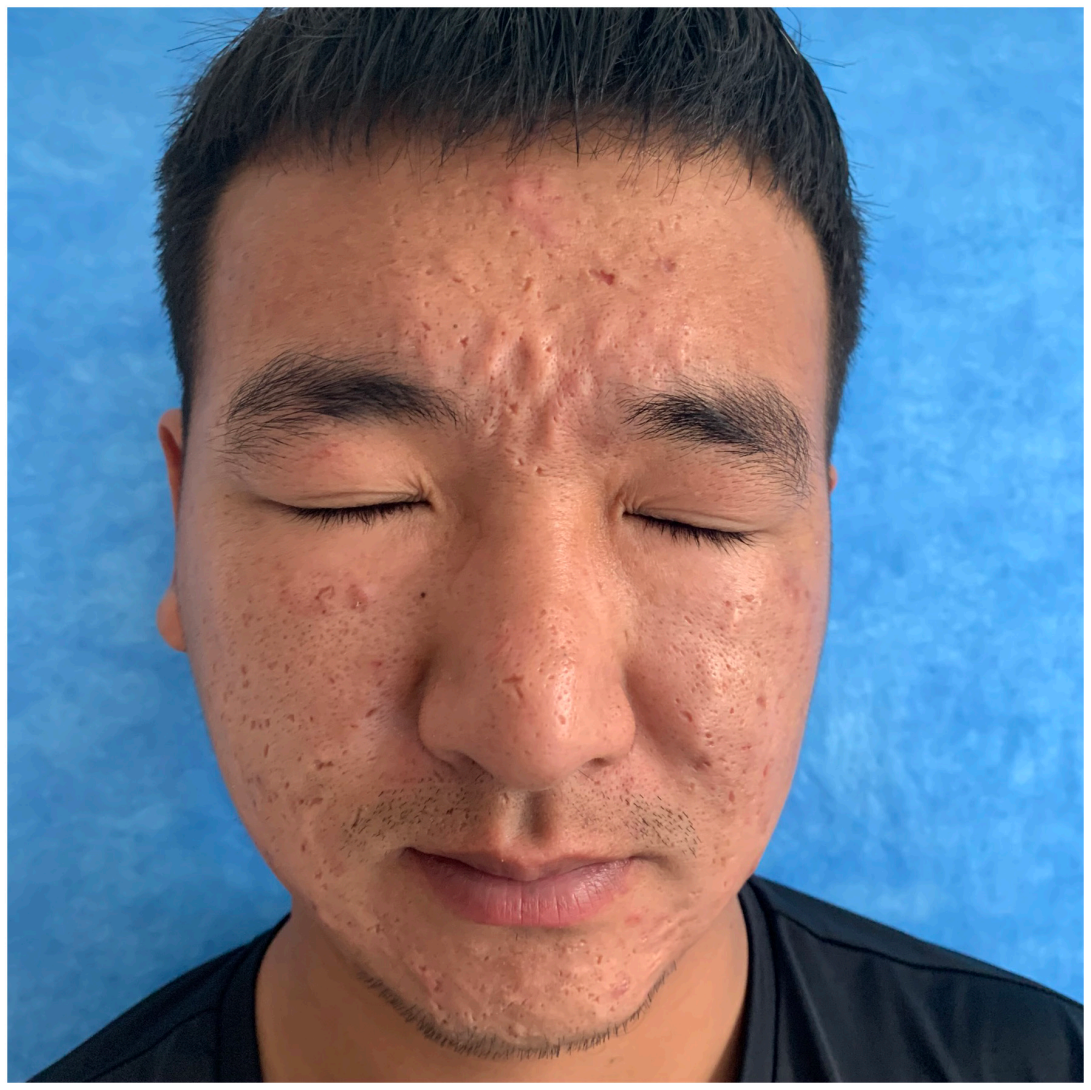
A 30-year-old male patient with frontal facial skin lesion.

**Figure 2:**
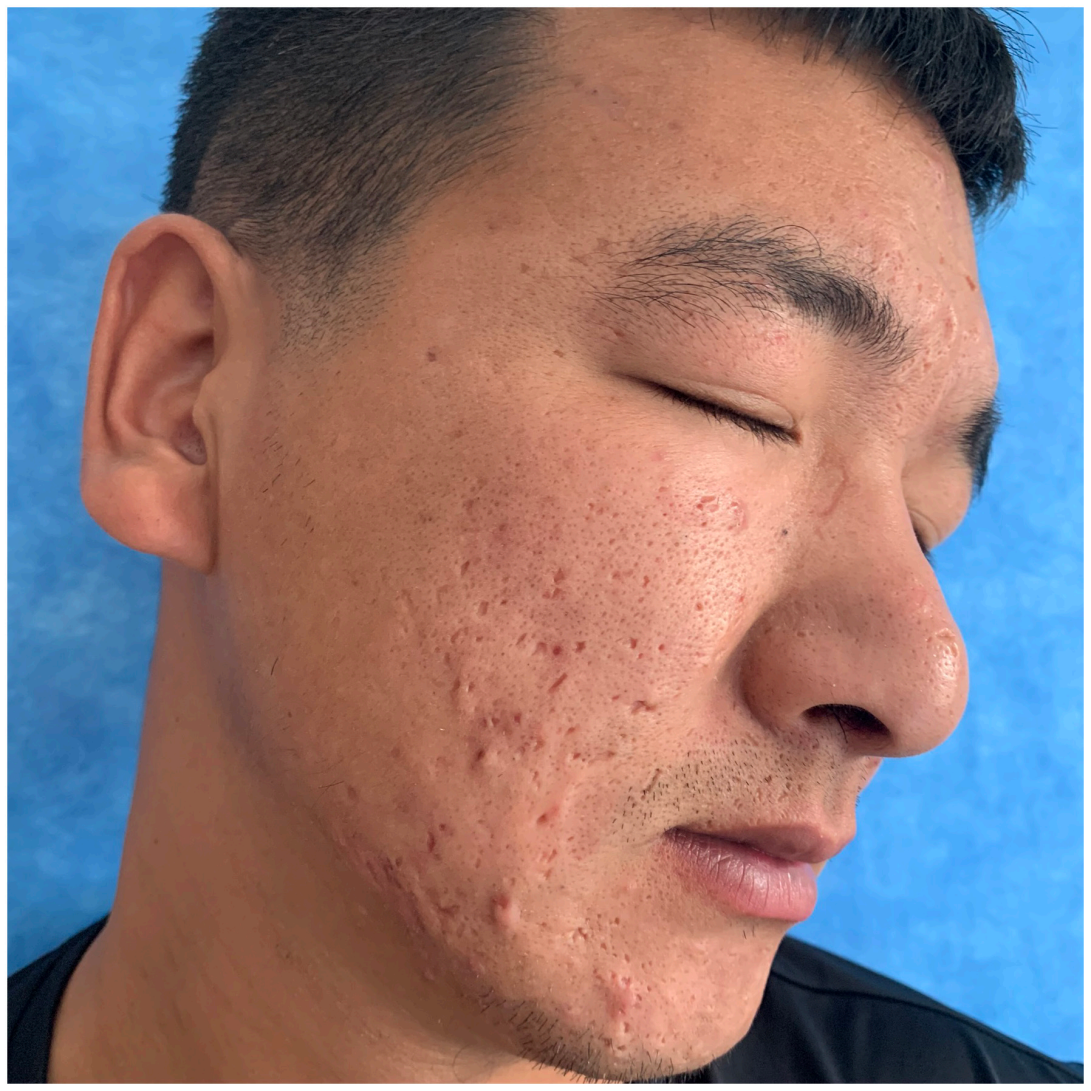
A 30-year-old male patient with right facial skin lesion.

Dermatological examination revealed multiple nodular acne lesions across the forehead, bilateral cheeks, and both mandibular areas, with a small number of inflammatory pustules scattered on both sides of the face. The face was covered with numerous atrophic scars of varying sizes, and it appeared rough, oily, and had enlarged pores (Figures 1, 2). No acneiform or folliculitis-like lesions were observed on the chest, back or scalp. After excluding other possible diagnoses, a diagnosis of occupational acne was established. The patient declined oral isotretinoin therapy and was therefore treated with oral minocycline in combination with topical adapalene cream and mucopolysaccharide polysulfate cream. He was also advised to change his occupation or increase protective measures at work. As the patient worked outside the local area, follow-up was conducted by phone call. He reported improvement in the inflammatory lesions after treatment; however, the nodular lesions showed no significant resolution. Because he remained in the same occupational environment, the acne continued to recur.

### 2.2 Case 2

A 45-year-old male electric welder reported the appearance of visibly enlarged facial pores and gradual eruptions of acne 6 months after he started working in electric welding 5 years earlier. His skin became prone to oiliness and his complexion turned darker. During the off-season periods of electric welding work when he engaged in farming at home instead, the acne lesions would significantly subside.

He had no history of acne during adolescence and none of his children have a history of acne. He reported no exposure to other industrial or chemical substances in either his occupational or living environment. He had no history of drug or cosmetic allergies and did not have a habit of using skincare products. His physical examination findings showed no evidence of autoimmune diseases or malignancies.

**Figure 3:**
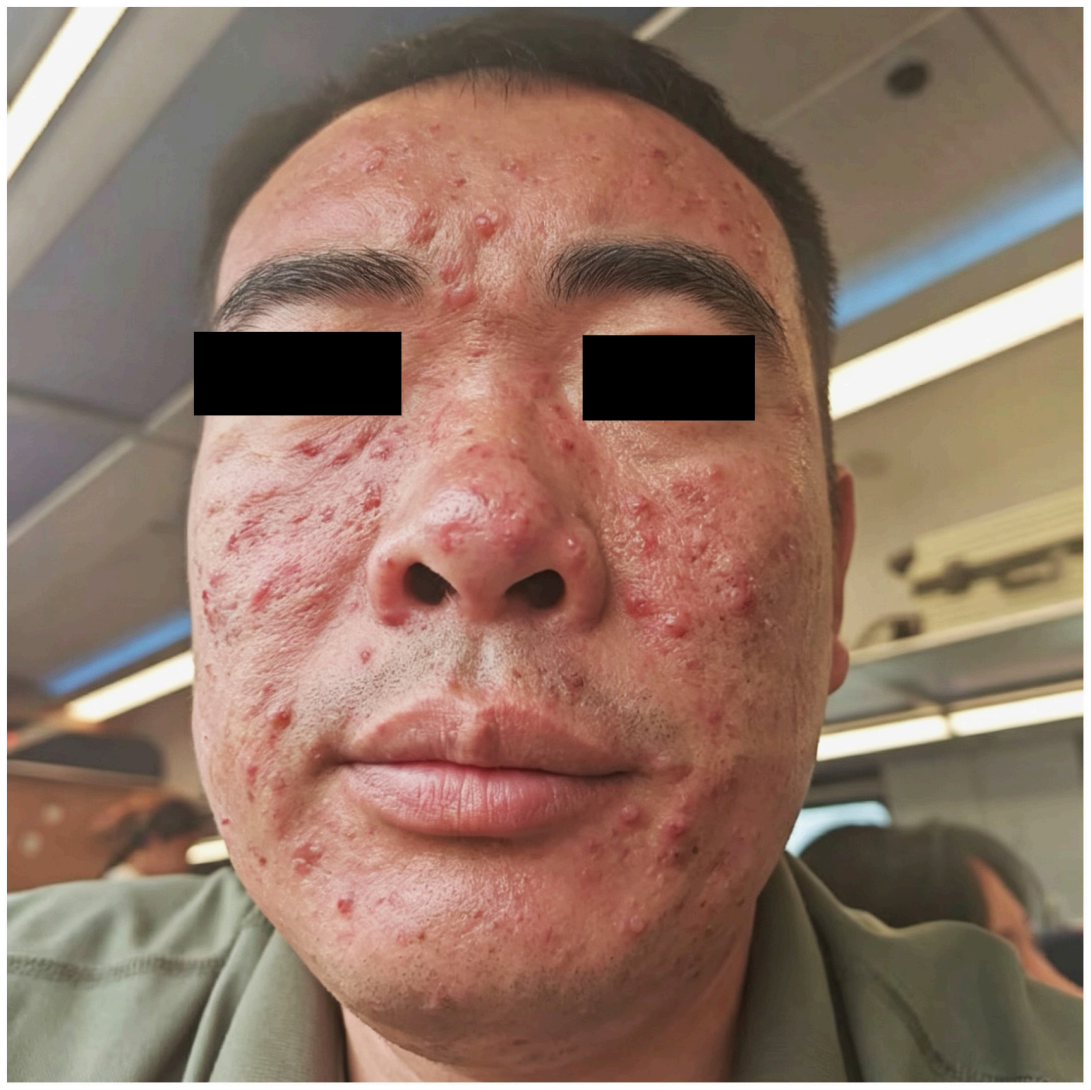
A 45-year-old male patient with facial skin lesion before treatment.

**Figure 4:**
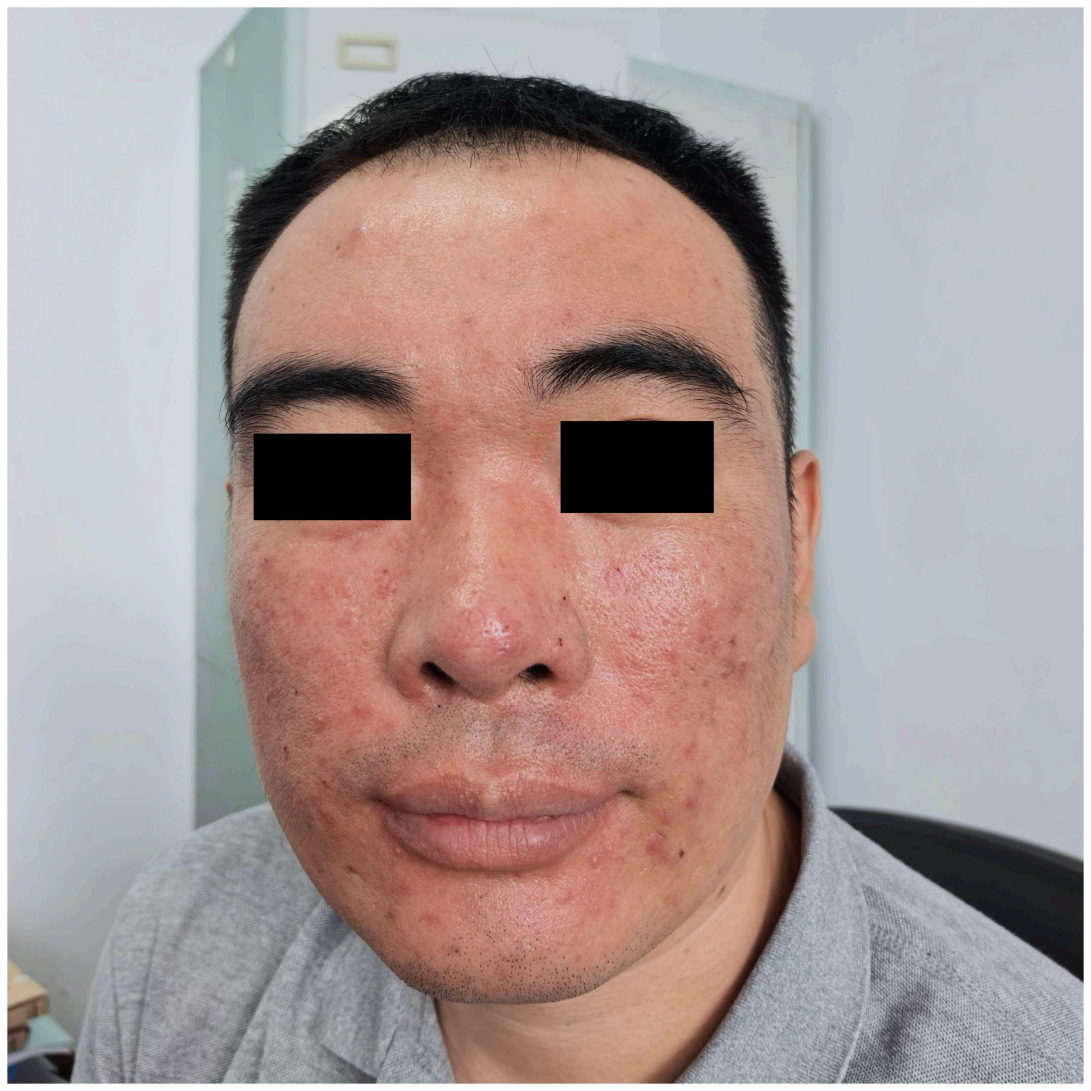
A 45-year-old male patient with facial skin lesion after treatment.

Currently, on examination multiple oval nodules were present on the forehead and temples, with both ends flat and the center elevated. They were firm and immovable. Similarly, multiple nodular acne were present on both cheeks and mandibular regions, with a large number of inflammatory pustules visible on both sides of the face. There were extensive atrophic scars of varying sizes as well on the face. (Figures 3–4).

No skin lesions were observed on the chest, back or scalp. Based on the overall clinical findings, a diagnosis of occupational acne was established. The patient was treated with oral minocycline in combination with topical adapalene cream, fusidic acid cream and mucopolysaccharide polysulfate cream. Following our recommendation, he was reassigned to an office-based position, thereby eliminating further occupational exposure. Follow-up was conducted online, during which the patient provided recent photographs demonstrating marked improvement in the acne lesions. Facial seborrhea and enlarged pores had also improved substantially. He reported only occasional acne flare-ups, which were adequately controlled with intermittent use of topical medications.

## 3 Discussion

During electric welding work, metal fumes, arc light, and high heat are generated, all of which contribute through several mechanisms leading to the development of acne.

### 3.1 Blockage of hair follicle openings induced by metal fumes

This is the primary and most direct cause.

- Physical blockage: during welding, a large amount of extremely fine metal oxide dust (e.g., iron, manganese, chromium, nickel, etc., depending on the welding rod and base material) and particles such as fluoride and silicate are released (2). These particles mix with sebum and sweat on the skin surface to form an “oil sludge”, which directly obstructs the hair follicle opening. This provides the key foundation for the initiation of exogenous acne.
- Chemical irritation: on the basis of physical blockage, certain metal particles such as chromium and nickel may irritate the skin (3), causing abnormal keratinization of the sebaceous gland ducts, further aggravating the blockage and stimulating inflammatory reactions.

### 3.2 Arc light

The arc light emitted during electric welding consists of infrared rays, visible light, and ultraviolet rays. Infrared rays have a thermal effect, and prolonged exposure to high-energy infrared rays may pose a risk of thermal damage, accelerate cell aging, and possibly induce skin inflammatory reactions (4). Among high-energy visible light, excessive exposure to blue light can exacerbate skin oxidative stress or oxidative damage (5), which are related to barrier damage and inflammation (6). For example, 460 nm blue light can penetrate 0.3 mm into the dermis, activate ROS-Clock genes, induce epidermal lipid peroxidation, and damage the barrier (7). Chronic exposure to ultraviolet rays can lead to photoaging and weaken the local immune defense function of the skin, thereby predisposing the skin to inflammation.

### 3.3 High-temperature environment

High-temperature environments are divided into sweltering environments caused by electric welding arc light and localized hot, humid environments on the face created by the use of protective equipment. High temperatures stimulate the sebaceous glands to secrete more oil, providing the “raw material” for the development of acne. At the same time, prolonged work and heavy sweating by workers not only exacerbate the hot and humid environment on the face, but also make it easier for salt, urea, and other components in sweat to mix with sebum and dust, forming blockages.

Both of the aforementioned patients were engaged in electric welding work, and their onset ages were in the late adolescent period, which is not a high-risk age for acne. Furthermore, neither of them had a prior history of acne during adolescence. During their vacations away from the work environment, their acne lesions significantly improved and new acne outbreaks decreased. These characteristics of onset are consistent with those of occupational acne and distinctly different from acne vulgaris. The differential diagnosis includes acne vulgaris, drug-induced acne, lupus miliaris disseminatus faciei, folliculitis, rosacea and contact dermatitis. These conditions can generally be distinguished based on the age at onset, medication history, characteristic clinical manifestations, lesion morphology, the presence and pattern of telangiectasia and a history of exposure to potential sensitizing agents.

Regarding the characteristics of the lesions, compared to the predominance of black comedones in oil acne and chloracne, this type of acne lesions primarily presented as nodules. This may be the result of the combined influence of various wielding-related pathogenic factors. Moreover, the lesions were commonly found on the forehead and bilateral mandibles, which were not shielded by protective masks. It is worth noting that despite the protection provided by hats, protective masks, and handheld face shields during welding, the forehead and bilateral mandibles remain exposed to the environment. Furthermore, handheld face shields can only block the light and heat in front of the worker, while light and heat generated by other workers during welding in the environment can still affect workers from different angles and directions.

We recommend improving workplace conditions in electric welding environments by installing adequate cooling and ventilation systems as well as exhaust devices for the removal of harmful gases. Electric welders should also be provided with more comprehensive personal protective equipment, including masks that protect not only the eyes from welding light but also the face and neck from occupational exposure. In addition, welders should cleanse the skin promptly after work to remove accumulated contaminants and may use measures such as cold compresses to reduce skin temperature. The use of skincare products or topical agents that help restore the skin barrier, as well as professionally formulated barrier creams for facial protection, may also be beneficial. Patients who develop acneiform eruptions should seek prompt medical evaluation and appropriate treatment to prevent disease progression and the formation of permanent disfiguring scars.

In summary, this type of acne associated with electric welding work merits utmost clinical attention and prompts us to consider new variants of occupational acne. Such patients are not uncommon in China, due to the large number of electric welding practitioners. We look forward to more cases from around the world and further research exploring the pathogenic effects of electric welding-related risk factors on the skin.

## Data Availability

The original contributions presented in the study are included in the article/Supplementary material, further inquiries can be directed to the corresponding author.
